# Special Issue “Structural and Physical Properties of Liquid Crystals”

**DOI:** 10.3390/ma19132719

**Published:** 2026-06-24

**Authors:** Marli Ferreira, Aloir Antonio Merlo

**Affiliations:** 1 Centre for Organic and Nanohybrid Electronics, Silesian University of Technology, Konarskiego 22b, 44-100 Gliwice, Poland; 2 Chemistry Institute, Federal University of Rio Grande do Sul, Bento Gonçalves 9500, Porto Alegre 91501-970, RS, Brazil

## 1. Introduction and Scope

Liquid crystals are a class of soft-matter systems that combine fluidity with long-range molecular order, leading to a wide range of optical, dielectric, and mechanical properties [1,2]. Their sensitivity to molecular structure and intermolecular interactions continues to make them an important platform for studying phase behavior and developing functional materials [3]. In this context, liquid-crystal research spans both fundamental investigations of mesophase structure and applied studies targeting responsive and composite systems [4,5,6].

This Special Issue of *Materials*, entitled “Structural and Physical Properties of Liquid Crystals”, highlights recent advances in the understanding of the relationships between molecular structure, supramolecular organization, and functional properties in liquid-crystalline materials. The collected contributions explore a broad range of topics, including chiral and frustrated liquid-crystalline phases—such as smectic glass-forming systems, twist–bend nematic phases, and cholesteric liquid-crystal elastomers—and blue phases, as well as dielectric anisotropy, ionic transport, and graphene–liquid-crystal hybrid systems.

The scope of this collection encompasses both fundamental and applied aspects of liquid-crystal science, integrating experimental, theoretical, and technological approaches to investigate phase behavior, molecular ordering, optical and dielectric properties, self-assembly, photoresponsiveness, and dynamic processes. Beyond fundamental investigations, three contributions highlight promising routes toward technological applications of liquid crystals, including light-responsive materials, flexible optical devices, and advanced sensing technologies [7,8,9].

The significance of this compilation lies in demonstrating how subtle molecular modifications and intermolecular interactions can strongly influence mesophase stability, electro-optical response, and material performance. Collectively, the articles provide important insights into the design of next-generation soft functional materials and highlight future perspectives for responsive photonic systems and multifunctional liquid-crystal-based technologies.

## 2. An Overview of the Published Articles

This Special Issue includes five original research articles and two review papers authored by researchers from research groups across China, Egypt, Greece, India, Japan, Poland, the Republic of Korea, Taiwan, and the United Kingdom, reflecting the global and collaborative nature of liquid-crystal research.

In the first article, “Partially Disordered Crystal Phases and Glassy Smectic Phases in Liquid Crystal Mixtures”, Deptuch et al. report that mixing the compound MHPOBC with the fluorinated compound 3F5FPhF6 provides an effective strategy for tuning phase behavior, crystallization, and glass formation in chiral smectic liquid crystals. All mixtures exhibited SmA*, SmC*, and SmCA* phases, whereas the hexatic SmXA* phase remained stable only at low fluorinated compound concentrations and disappeared as molecular disorder increased. The equimolar mixture displayed the highest glass-forming ability, completely avoiding crystallization during cooling, while other compositions underwent partial crystallization into orientationally disordered crystal phases. Structural and dielectric investigations revealed a gradual decrease in positional order with increasing fluorination and fragile glass behavior characterized by super-Arrhenius α-relaxation. Using a combination of DSC, POM, XRD, and broadband dielectric spectroscopy, the authors correlated phase transitions, molecular organization, and relaxation dynamics across different compositions. The results highlight the competition between ordered smectic phases and vitrified states, showing that increasing molecular disorder promotes glass formation while suppressing highly ordered smectic phases, and provide valuable guidelines for the design of liquid-crystal systems with tailored electro-optical properties.

In the second article, “Solvent Evaporation-Induced Self-Assembly of Flexible Cholesteric Liquid Crystal Elastomers: Fabrication, Performance Tuning, and Optimization”, Zhang et al. present a simple and efficient SEISA method for fabricating flexible double-layer cholesteric liquid-crystal elastomers (DCLCEs) with broadly tunable structural colors. They address important limitations of conventional CLCE systems, including complex fabrication procedures, limited flexibility, bubble defects, and restricted optical response. The results demonstrate that both optical and mechanical properties can be controlled through optimization of fabrication parameters: by adjusting the concentration of the chiral dopant LC756, the authors tuned the structural color from red to blue, controlling the helical pitch and reflected wavelength range. The optimized materials exhibited a mechanochromic response, with the reflection wavelength shifting from 613 nm to 404 nm under stretching, corresponding to a tunable bandwidth of 209 nm across the visible spectrum. In addition, controlling the solvent evaporation temperature between 20 and 30 °C minimized bubble formation, improving film homogeneity and color purity. Mechanical performance was further optimized through UV photopolymerization, with irradiation times between 180 and 220 s balancing flexibility and crosslinking density. Blade-coating parameters, particularly a blade gap of 150–200 μm and coating speed of 20 mm/s, enabled uniform films with excellent monochromaticity. Overall, the work establishes the SEISA method as a scalable strategy for flexible photonic materials with tunable structural colors for applications in flexible displays, wearable sensors, smart coatings, anti-counterfeiting technologies, and adaptive optical devices.

In the third article, “Overlooked Ionic Contribution of a Chiral Dopant in Cholesteric Liquid Crystals”, Shaban et al. demonstrate that ionic contributions originating from chiral dopants in cholesteric liquid crystals (CLCs) are often overlooked, despite their strong influence on electro-optical behavior. Using dielectric spectroscopy, the authors investigated E44-based CLC systems doped with the enantiomeric chiral dopants R5011 and S5011 and identified significant differences in their ionic responses. While the left-handed dopant S5011 produced almost no noticeable ionic contribution, the right-handed dopant R5011 introduced a substantial concentration of mobile ions into the liquid-crystal matrix. With increasing concentration of the latter, dielectric loss, ion density, ion diffusivity, and DC conductivity increased markedly, indicating enhanced ionic transport, whereas S5011-doped samples remained similar to pure E44. The ion density in E44/R5011 systems increased by approximately 70%, confirming the dopant as a major ion source. Thermal analysis showed Arrhenius-type behavior for ionic diffusivity and conductivity. Moreover, binary racemic mixtures exhibited ionic properties similar to those of R5011 systems, confirming its dominant contribution. The study highlights the importance of dopant purity and ionic contamination for device stability and performance, while also suggesting that controlled ionic transport may be exploited for tunable optical and electro-optical applications.

In the fourth article, “Photoinduced Phase Transitions of Imine-Based Liquid Crystal Dimers with Twist-Bend Nematic Phases”, Arakawa et al. report the synthesis of two homologous series of thioether-linked liquid-crystal dimers containing inverse ester linkages, namely CBCOO*n*SBA(CN) and CBOCO*n*SBA(CN), and demonstrate reversible photoinduced phase transitions in imine-based twist–bend nematic (NTB) liquid crystals. Both series exhibit monotropic NTB phases below the conventional nematic phase, and selected homologs show supercooling and vitrification of the NTB phase near room temperature. The results indicate that molecular geometry strongly affects liquid-crystal behavior, with COO-linked dimers showing lower isotropic-to-nematic transition temperatures and greater molecular bending than OCO-linked analogs, reflecting stronger molecular biaxiality. Under UV irradiation at 365 nm, reversible phase transitions from the nematic to the isotropic phase (N → Iso) and from the NTB to the nematic phase (NTB → N) were observed, with rapid recovery after switching off the light. The photoresponse is attributed to imine bond photoisomerization, supported by UV–Vis absorption around 370 nm, although the operational temperature window remains limited due to the higher activation barrier compared with azobenzene systems. Overall, the work highlights imine-based NTB dimers as promising light-responsive materials for photonic devices, optical switches, and actuators, and clarifies the relationship between molecular structure and NTB phase stability.

In the fifth article, “Comparative Study of the Optical and Dielectric Anisotropy of a Difluoroterphenyl Dimer and Trimer Forming Two Nematic Phases”, Zavvou et al. investigate the influence of molecular architecture on the optical and dielectric properties of Nx-forming liquid-crystal oligomers. By comparing a monomer, dimer, and homologous trimer based on laterally fluorinated difluoroterphenyl units, the authors provide important insight into how the number of mesogenic units affects orientational order, dipolar correlations, and nematic phase behavior. The results show that the dimer and trimer possess remarkably similar optical and dielectric properties despite their different molecular sizes. The monomer exhibited the highest birefringence, whereas the dimer and trimer displayed lower but comparable values, indicating similar orientational order within the oligomeric systems. Upon cooling within the nematic phase, birefringence increased but deviated from classical Haller behavior near the N–Nx transition, where saturation effects became evident. After entering the Nx phase, birefringence decreased due to the heliconical tilt of the mesogenic units. Dielectric studies revealed weakly negative dielectric anisotropy for both oligomers, with magnitudes lower than that of the monomer but nearly identical between the dimer and trimer. An important conclusion is that lateral fluorine substituents promote parallel intermolecular dipolar correlations perpendicular to the nematic director, generating local ferroelectric-like ordering. Overall, the work expands the understanding of structure–property relationships in liquid-crystal oligomers and provides guidelines for designing materials with tunable optical and dielectric responses.

In the first review paper, “Graphene–Liquid Crystal Synergy: Advancing Sensor Technologies across Multiple Domains”, Adeshina et al. discuss the growing importance of combining graphene with liquid crystals to create highly sensitive and multifunctional sensing platforms. The significance of the work lies in demonstrating how graphene’s exceptional electrical, thermal, optical, and mechanical properties can be integrated with the self-organizing and stimuli-responsive nature of liquid crystals to improve sensor performance across several technological fields. The article highlights advances in thermal and infrared sensing, flexible actuators, chemical and biological detection, and environmental monitoring. Reduced graphene oxide-doped liquid crystals exhibited efficient photothermal responses suitable for infrared detection and thermal mapping. In flexible and wearable systems, graphene-enhanced liquid-crystal elastomers displayed reversible shape-memory behavior and responsive actuation under light and heat, and in chemical and biological sensing, graphene oxide/liquid-crystal systems enabled sensitive detection of biological interactions and drug monitoring. The review emphasizes the broad, interdisciplinary scope of graphene–liquid-crystal systems and their superior sensitivity and adaptability compared with conventional sensor technologies. From a broader perspective, the authors highlight challenges related to scalability, cost, and integration, while pointing to strong future potential for wearable electronics, smart textiles, and multifunctional sensing devices.

In the second review paper, “Amorphous Blue Phase III: Structure, Materials, and Properties”, Yoshizawa reviews Blue Phase III liquid crystals, which are highly attractive for next-generation display technologies because they operate without surface alignment layers, switch within submilliseconds, and provide wide viewing angles. However, this phase has historically been unstable for practical applications, existing only within a very narrow temperature range of approximately two kelvin. The review provides significant insight into the three-dimensional structure of Blue Phase III and establishes molecular design principles for stabilizing the phase at room temperature. Advanced imaging techniques and computational simulations reveal that the phase consists of a highly fluid, spaghetti-like tangled arrangement of double-twist cylinders rather than a rigid cubic lattice. The author demonstrates that stabilization requires molecules with both high biaxiality and high structural flexibility, enabling liquid-like disorder. Various strategies are discussed, including ternary mixtures, polymer network confinement, quantum dot doping, and specialized molecular architectures. The work also examines nanoscale memory effects and highlights how polymer scaffolds can transfer structural templates into achiral liquids. Although substantial progress has been achieved, the main limitation remains the high operating voltage required for device applications, with future developments focusing on molecular engineering approaches to reduce driving voltages for practical ultra-fast display technologies.

## 3. Future Perspectives and Outlook

Recent advances in liquid-crystal science have expanded the range of accessible mesophases and functional responses; however, important challenges remain in achieving a full molecular-level understanding of structure–property relationships, particularly in systems involving chirality, frustration, and nonequilibrium dynamics. Despite substantial progress, predictive control over phase stability, defect structures, ionic effects, and dynamic processes is still limited [10,11], especially in complex and hybrid systems [12].

Future research should focus on integrating multiscale modeling with advanced experimental techniques to develop predictive frameworks for liquid-crystal behavior. Increasing attention should also be given to nonequilibrium processes, defect engineering, and reconfigurable systems. Emerging approaches such as machine learning-guided design [13], active liquid crystals, and hybrid soft-matter architectures are expected to further advance the field [12]. Finally, translation into scalable and stable devices remains essential for applications in photonics, sensing, and adaptive technologies [14,15].

## 4. Conclusions

This Special Issue demonstrates the vibrant and ever-changing field of liquid-crystal research. The varied collection of original research and review articles included here effectively connects basic questions about molecular organization with the creation of next-generation functional materials. By revealing new design principles and mechanisms, these contributions improve our basic understanding and provide a clear path for future technology applications. As Guest Editors, we hope this volume acts as a helpful resource and inspires further innovation within the soft-matter community.
